# Insights Into the Roles of GATA Factors in Mammalian Testis Development and the Control of Fetal Testis Gene Expression

**DOI:** 10.3389/fendo.2022.902198

**Published:** 2022-05-26

**Authors:** Robert S. Viger, Karine de Mattos, Jacques J. Tremblay

**Affiliations:** ^1^Centre de recherche en Reproduction, Développement et Santé Intergénérationnelle and Department of Obstetrics, Gynecology, and Reproduction, Faculty of Medicine, Université Laval, Quebec City, QC, Canada; ^2^Reproduction, Mother and Child Health, Centre de recherche du centre hospitalier universitaire de Québec—Université Laval, Quebec City, QC, Canada

**Keywords:** transcription, GATA, hormone, testis, fetal, gene expression

## Abstract

Defining how genes get turned on and off in a correct spatiotemporal manner is integral to our understanding of the development, differentiation, and function of different cell types in both health and disease. Testis development and subsequent male sex differentiation of the XY fetus are well-orchestrated processes that require an intricate network of cell-cell communication and hormonal signals that must be properly interpreted at the genomic level. Transcription factors are at the forefront for translating these signals into a coordinated genomic response. The GATA family of transcriptional regulators were first described as essential regulators of hematopoietic cell differentiation and heart morphogenesis but are now known to impact the development and function of a multitude of tissues and cell types. The mammalian testis is no exception where GATA factors play essential roles in directing the expression of genes crucial not only for testis differentiation but also testis function in the developing male fetus and later in adulthood. This minireview provides an overview of the current state of knowledge of GATA factors in the male gonad with a particular emphasis on their mechanisms of action in the control of testis development, gene expression in the fetal testis, testicular disease, and XY sex differentiation in humans.

## Introduction

### The Vertebrate Family of GATA Transcription Factors

The GATA family of transcriptional regulators comprises six factors (GATA1 to 6) in vertebrates. GATA factors regulate gene transcription by binding to a consensus nucleotide sequence—A/TGATAA/G—called the GATA motif that is scattered throughout the genome. The six GATA proteins are typically classified based on their amino acid sequence and tissue distribution patterns. GATA1/2/3 are hallmarks of hematopoietic cell lineages, and consequently are essential for the differentiation of multiple blood cell types (erythrocytes, leukocytes, megakaryocytes) [reviewed in (1, 2)]. By contrast, the GATA4/5/6 proteins (known as the cardiac family of GATA factors) populate tissues of mesodermal and endodermal origin such as the heart, organs of the digestive tract, and gonads [reviewed in (1–5)]. Initial insights into the cardiac subfamily of GATA factors came from global gene inactivation studies in mice. Loss of GATA4 precipitates early embryo lethality because of defects in ventral morphogenesis of the embryo and its subsequent inability to form a functional linear heart tube (6, 7). Lack of *Gata6* expression arrests embryo development at an even earlier stage due to defects in extraembryonic development (8). Unlike GATA4 and GATA6, global loss of GATA5 function is not embryonic lethal; rather *Gata5^-/-^* mice exhibit prominent genitourinary tract abnormalities in females but not males (9). Identification of *GATA* gene mutations associated with clinical disease has helped to highlight the essential nature of GATA function in humans. For the *GATA4* gene alone, more than 100 mutations have been linked to a large spectrum of cardiac abnormalities [reviewed in (2, 10)].

All GATA proteins share a highly conserved zinc finger DNA-binding domain. The specificity of GATA action is conferred in part through interactions with specific partners [reviewed in (2–4)]. The most notable are the Friend of GATA (FOG) proteins [reviewed in (11)]. GATA factors also interact and functionally cooperate with a multitude of other transcription factors that not only amplify GATA activity but also limit their scope of action to select target genes. This is true for the testis where many potential GATA-interacting partners have been identified (12–18). GATA activity can also be positively or negatively modulated by direct post-translational modifications such as sumoylation, acetylation, and phosphorylation. Post-translational modifications alter GATA4 transcriptional activity by changing either its nuclear localization, DNA-binding, stability, and/or cofactor recruitment [reviewed in (10, 19)].

## GATA Factors in the Testis

### Developmental and Cell Type-Specific Expression Patterns

Three GATA factors (GATA1/4/6) are found in the testis and their expression profiles differ based on testicular cell type and timing of expression. *Gata1* was the first reported to be abundantly transcribed in the testis (20). It is also the only GATA factor limited to a single testicular cell type: the postnatal Sertoli cell (21). GATA1 is absent from the fetal testis and as such is not involved in early testis development (22). The onset of *Gata1* expression in mouse Sertoli cells coincides with the first wave of spermatogenesis; thereafter GATA1 levels remain constant and independent of hormones but become progressively spermatogenic stage-dependent (21, 22). GATA1 is therefore an excellent marker of mature Sertoli cells; however, its functions appear to be dispensable for Sertoli cells, at least in mice (23). GATA4 is one of the earliest markers of gonadal development (22, 24). Expression begins in the coelomic epithelial layer of the presumptive genital ridges of both the mouse and chick (24–26). After testis differentiation, GATA4 levels are upregulated in Sertoli, myoid, and Leydig cells [(22, 26, 27) and reviewed in (28)]. High GATA4 levels persist throughout fetal development and into adulthood (22, 27). Like *Gata4*, the *Gata6* gene is transcribed in Sertoli cells of both fetal and adult testes (29, 30). However, unlike *Gata4*, Sertoli cell *Gata6* transcripts only become upregulated during the later fetal stages (29, 30). While *Gata6* expression has been documented in at least one Leydig cell line (29), data showing that the gene is also expressed in Leydig cells *in vivo* is less robust. A review article published in 2014 citing unpublished observations nonetheless reported that GATA6 is present in both late fetal and postnatal Leydig cells in the mouse (5).

### Roles in Early Mammalian Gonad Development and Male Sex Determination

Of the three GATA factors expressed in testis, only GATA4 is involved in early gonad development—in the absence of GATA4, coelomic thickening that permits the subsequent growth of the genital ridge is arrested (24). In the same study, the authors also demonstrated that GATA4 acts early being upstream of LHX9 and SF1, two other critical regulators of genital ridge development (31, 32). GATA4 function in gonadal development does not end at the genital ridge stage as revealed by elegant gene inactivation studies in mice, which showed that GATA4 continues to play multiple essential roles with the timing of *Gata4* deletion being critical to the phenotypic outcome [reviewed in detail in (5)]. *Gata4* deletion prior to sex determination completely blocks testis differentiation and results in XY sex reversal (33). By contrast, *Gata4* deletion after male sex determination allows testis differentiation to proceed unfettered (33). In this same model, testis cord development is nonetheless disrupted due to diminished *Dmrt1* expression (described more below), and males are undervirilized and eventually become infertile (33). GATA4 action is also required in the postnatal testis where it controls male fertility and steroidogenesis (34–39). Its role in male fertility appears to be linked to regulation of blood-testis barrier integrity and Sertoli cell genes critical for this function (35, 40).

GATA4 initiates testis differentiation by regulating at least two genes: Sex determining region Y chromosome (*Sry*) and Sry-related homeobox gene 9 (*Sox9*); both genes are markedly downregulated in newly differentiated testes that lack a fully functional GATA4 protein (41–43). However, as for many of the proposed GATA target genes in the testis, it remains to be seen whether they are indeed direct targets. For the *Sry* gene, evidence suggests that this might be the case. A detailed analysis of *Sry* 5’ flanking sequences from 17 mammalian species identified a single broadly conserved 106 bp region termed the *Sry* proximal conserved interval (SPCI). The SPCI contains a conserved binding motif for WT1, a known activator of both mouse and human *SRY* transcription (44–46). WT1 has also been reported to directly cooperate with GATA4 on the mouse, pig, and human *SRY* promoters (15). The requirement for GATA4 in *Sry* and *Sox9* transcription has raised the important question as to whether it also participates in the specification of the Sertoli cell lineage at the time of sex determination. Mice lacking a functional GATA4 protein that can no longer interact with FOG2 do not express any Sertoli cell markers (41), indicating that the GATA4-FOG2 complex (and by extension GATA4 itself) is required for the differentiation of pre-Sertoli cells. Additional insights have come from studies that have reprogrammed fibroblasts into embryonic Sertoli cells. Initial studies showed that GATA4 along with other transcription factors (SF1, WT1, DMRT1, SOX9, DMRT1) are sufficient to induce the formation of embryonic Sertoli-like cells (47–49). A later study refined the mechanism of GATA4 induction of the Sertoli cell fate by showing that it acts upstream of SF1, LHX9, and the anti-Müllerian hormone (AMH) (50).

## GATA Factors in Fetal Testis Gene Expression and Function

### GATA Control of Fetal Sertoli Cell Gene Expression

Following sex determination, the newly formed testis (by producing key fetal hormones) assumes several important functions in masculinizing the developing male embryo. GATA factors continue to play an important role in regulating genes that participate in this fundamental developmental process. Sertoli cells begin to produce AMH, a glycoprotein hormone belonging to the transforming growth factor beta (TGFβ) superfamily. AMH is most recognized for its role in blocking the Müllerian ducts from developing into female reproductive structures in typical XY males (51). The *AMH* promoter from multiple species contain at least one consensus GATA motif that is activated by GATA4 (22, 52–55). In cell lines, the mechanism of GATA4 action on *AMH* transcription has been proposed to involve synergistic interactions with other transcription factors including SF1 and WT1 (12, 15, 56). A more recent *in vivo* study that inactivated the GATA regulatory motif in the *Amh* promoter in the mouse has validated not only the *Amh* gene as a direct target for GATA4 but also the requirement of a GATA4/SF1 interaction for *Amh* expression in fetal Sertoli cells (57). Endogenous *Amh* expression is also controlled by direct binding of SF1 and SOX9 to the *Amh* promoter (58). Unlike SOX9, however, which is required to initiate the production of *Amh* transcripts in newly differentiated Sertoli cells, GATA4 appears to function like SF1 as an amplifier of *Amh* transcription (57, 58).

While mutagenesis of the *Amh* promoter has highlighted the requirement of GATA binding for *Amh* transcription, simultaneous deletion of *Gata4* and *Gata6* in Sertoli and Leydig cells in the fetal testis has favored the opposite view (38). GATA1 was first proposed to disrupt GATA4 action at the *Amh* promoter thereby decreasing *Amh* expression (59). A separate study reported that AMH levels remained high beyond its normal period of decline in postnatal mouse testis lacking both GATA4 and GATA6 (38). GATA1 was also absent in this model (38). These observations led to the suggestion that GATA factors negatively regulate *Amh* gene transcription, at least in postnatal Sertoli cells (38). However, male gonads lacking GATA4 and GATA6 also have compromised steroidogenic gene expression during the fetal period, and a reported reduction of serum testosterone beyond puberty (38). Testosterone, acting through the androgen receptor, is known to attenuate AMH production in Sertoli cells (60, 61). This might account for the differences in *Amh* expression seen in a GATA4/6-deficient mouse testis model where androgen production is notably reduced (38), in comparison to a mouse model where the GATA binding motif in the *Amh* promoter is mutated and androgen levels are not affected (57).

The *Dmrt1* gene has been proposed to be another GATA4 target in the fetal testis (33). DMRT1 is an evolutionarily conserved transcription factor that regulates testis development in all vertebrates (62). Testes from *Dmrt1^-/-^* mice, and animals where *Gata4* was conditionally inactivated in testes after sex determination, share a similar testicular cord defect beginning at the mid-fetal stage (33). This suggested that *Dmrt1* gene expression in Sertoli cells is directly regulated by GATA4. In support of this, the *cis*-regulatory elements for the *Dmrt1* gene have been mapped in primary Sertoli cells cultures and shown to contain multiple active GATA binding motifs (63). However, GATA4 is not required to maintain somatic *Dmrt1* expression after fetal development as adult Sertoli cells lacking GATA4 continue to express *Dmrt1* (33). Moreover, while both GATA4 and FOG2 are required for the differentiation of pre-Sertoli cells, *Dmrt1* expression in fetal Sertoli cells continues to require GATA4 but not FOG2 (33).

Several other Sertoli cell-expressed genes have been proposed to be regulated by GATA factors. These include inhibin α (*Inha*), inhibin β B (*Inhbb*), reproductive homeobox 5 (*Rhox5*), and follicle stimulating hormone receptor (*Fshr*) (14, 29, 64–69). The GATA-dependence of these additional putative GATA targets have been limited to promoter activation assays and studies where GATA levels have been manipulated in immortalized cell lines. Whether these genes are in fact genuine GATA targets *in vivo* and what contribution GATA factors have to these genes in terms of fetal Sertoli cell expression remain unanswered.

### GATA Control of Fetal Leydig Cell Differentiation and Gene Expression

Leydig cells regulate male sex differentiation during fetal development, and fertility in adults, through the production of two hormones: insulin-like 3 (INSL3) and testosterone. INSL3 initiates the first of two phases of testicular descent during embryogenesis (70, 71). Testosterone is a steroid hormone made from the multistep enzymatic conversion of cholesterol. Testosterone regulates the second phase of testis descent and promotes the masculinization of the Wolffian ducts into organs of the male reproductive tract. In mammals, there are two distinct populations of Leydig cells: fetal Leydig cells (FLCs) present during fetal life and shortly after birth, and adult Leydig cells (ALCs) present in the postnatal testis. FLCs are proposed to derive from progenitor cells originating within the adreno-gonadal primordium and other sources within the mesonephros, neural crest cells, coelomic epithelium [reviewed in (72)]. Many transcription factors have been implicated in FLC differentiation and function [reviewed in (73, 74)]. GATA4 appears to play an integral role in both of these processes. First, XY *Gata4^−/−^* embryonic stem (ES) cells fail contribute to FLCs in chimeric mouse embryos (75). Second, teratomas derived from XY *Gata4*^++^, but not XY *Gata4*^−/−^ ES cells, express steroidogenic markers when grown in gonadectomized nude mice (75). Together, these two experimental models provided the first evidence that GATA4 is likely required for the cell autonomous differentiation of FLCs. XY fetuses lacking GATA4 in testes at the time of male sex determination are undervirilized with partially descended testes but whose steroidogenic function is apparently intact (33). A major reduction in the number of cells expressing P450 side chain cleavage enzyme (CYP11A1) and 3β-hydroxysteroid dehydrogenase (3βHSD) only occurs in the testicular interstitium of mice lacking both GATA4 and GATA6 (38), suggesting that FLC differentiation and/or their steroidogenic function requires both factors. These mice also have reduced *Insl3* expression; no other *in vitro* or *in vivo* data exists, however, that would suggest the *Insl3* gene to be a direct transcriptional target for GATA factors. In this same model, steroidogenic expression observed in postnatal testes was notably increased but was accompanied by a significant decrease in circulating testosterone (38). The authors of this study further showed that the effects on postnatal testicular steroidogenesis and androgen production were not driven from FLCs or ALCs but rather from an expansion of adrenocortical-like cells residing in the testicular interstitium—thus making the direct contribution of GATA4/6 to Leydig cell gene expression and function in this model difficult to assess.

Independent groups have postulated that GATA4 functions as a master regulator of Leydig cell steroidogenesis based on data generated in steroidogenic cell lines (34, 36, 76). Blockade of *Gata4* expression in Leydig cell lines leads to a suppression of the steroidogenic program and a decrease in hormone production (34, 76). Loss of both GATA4 and GATA6 in primary Leydig cells (77), or disruption of their transcriptional activities in mice (36), has also been shown to suppress steroidogenesis by reducing the expression of several genes involved in androgen biosynthesis, including steroidogenic acute regulatory protein (*Star*), *Cyp11a1*, 3β-hydroxysteroid dehydrogenase 1 (*Hsd3b1*), 17α-hydroxylase (*Cyp17a1*), and 17β-hydroxysteroid dehydrogenase 3 (*Hsd17b3*)—many of these genes have conserved GATA motifs in their *cis*-regulatory regions [reviewed in (4, 18)]. Since some of these genes are also reduced in fetal testes lacking GATA factors but only when the *Gata4* and *Gata6* genes are simultaneously deleted, both factors likely compensate for one another in regulating FLC gene expression. Transcription of several other Leydig cell-expressed genes have been proposed to be targeted by either GATA4 and/or GATA6 [reviewed in (4, 18)]. The GATA-dependence of these additional targets has been mostly limited to characterization of their *cis*-regulatory regions. Therefore, their validation as genuine *in vivo* and/or direct targets of GATA factors remains to be determined.

## Regulation of GATA Factors in the Fetal Testis

The regulation of *GATA* gene expression and GATA transcriptional activity in the fetal testis remains poorly understood. The transcriptional regulation of the *Gata4* gene was first reported in 2006 (78). In that initial study, activity of the first 124 bp upstream of the mouse *Gata4* transcriptional start site in Leydig and Sertoli cell lines was shown to be dependent on conserved GC-rich and E-box elements (78). Similar findings highlighting the importance of GC-rich and E-box elements were later reported in context of 5 kb of the rat *Gata4* promoter (25). The same 5 kb of rat *Gata4* promoter was also shown to be sufficient to drive GFP expression in the male gonad of transgenic mice from the onset of testis differentiation to adulthood (25). Subsequent analysis showed high transgene expression in both Sertoli and Leydig cells, and at all times (both fetal and mature testis) (79). Targeted mutagenesis of the *Gata4* E-box element in the mouse confirmed the importance of this motif for endogenous *Gata4* expression in both fetal and adult testis (80). Like other *GATA* genes, the rodent and human *GATA4* genes are expressed as multiple transcripts that differ in their 5’ untranslated exons, which are driven by alternative promoters (81). Thus, in addition to the typical *Gata4* transcript driven by the E-box dependent proximal *Gata4* promoter, the fetal testis also expresses a second *Gata4* transcript driven by an alternative promoter sequence located nearly 30 kb upstream of the proximal promoter (81, 82). The distal *Gata4* promoter appears to be autoregulated by GATA4 itself in a regulatory mechanism proposed to ensure sufficient *Gata4* expression in the testis at critical times such as during testis differentiation (82). Characterization of the *cis*-regulatory regions required for *Gata6* expression in the testis have not been reported.

Despite their profound roles in testis physiology, the hormonal regulation of GATA factors in the testis have received comparably little attention. Nonetheless, treatment of Sertoli and Leydig cell lines with gonadotropins causes a modest increase in *Gata4* expression (29, 83). Gonadotropins and androgens, however, do not appear to be required for basal *Gata4* or *Gata6* expression in the testis as their expression is retained in different models that perturb these hormones (29). In contrast to the paucity of information on hormones that regulate GATA factors in the testis, much more is known about the signaling pathways activated following hormone stimulation and their impacts on the transcriptional activity of GATA factors, especially GATA4. Two GATA4 phosphorylation sites have been well described: serine 105 (S105) targeted by mitogen-activated protein kinases (MAPK) and serine 261 (S261) targeted by protein kinase A (PKA) (84–87). In the testis, GATA4 phosphorylation by PKA is particularly informative since it helps to better understand the acute gonadotropic regulation of multiple testis-expressed genes that lack classic cAMP response elements (CREs), but contain GATA regulatory motifs, in their respective promoters. For both S105 and S261, *in vitro* studies have shown that GATA4 phosphorylation stimulates GATA4 transcriptional activity on multiple testis target genes (87, 88). GATA4 S105 phosphorylation by MAPK has also been proposed to be essential for GATA4 action in the upregulation of *Sry* expression during testis differentiation (89, 90). A study published in 2019, addressed the *in vivo* role of GATA4 phosphorylation through the detailed characterization of two mouse models (GATA4 S105A and GATA4 S261A) that carry mutations that specifically block GATA4 phosphorylation at either site. One of these mutations (S105A) was associated with a male hormonal defect characterized by a deficiency in testosterone production in adult but not fetal males (36). Interestingly, male sex determination was unaffected in GATA4 S105A mutants (36), which questions the requirement for GATA4 phosphorylation (at least on S105) in the testis differentiation process.

## GATA Factors in Human Testis and Testicular Pathologies

Several studies have investigated the expression and potential role of GATA factors in normal human testis development and function, as well as the pathogenesis of testicular tumors [(91–94) and reviewed in (95)]. Much like rodents and other mammalian species, GATA4 and GATA6 appear to be the predominant GATA factors of the human fetal testis. In Sertoli cells, GATA4 is present during both fetal and postnatal development (91). *GATA4* is also expressed in fetal and postpubertal Leydig cells which correlates with its proposed role in steroidogenesis (91). Interestingly, *GATA4* expression is significantly higher in both Sertoli and Leydig cell tumors (91), suggesting that GATA4 might influence cell proliferation during tumorigenesis in human testicular somatic cells. Unlike rodents, GATA4 is a marker of a subset of early fetal gonocytes (91), as well as multiple testicular germ cell neoplasia that include both precursor carcinoma *in situ* (CIS) testis cells and definite testicular germ cell tumors (seminomas, yolk sac tumors, teratomas) (93). Testicular seminomas and yolk sac tumors also express *GATA6* (93). The presence of GATA4 and/or GATA6 in these cells has suggested a role for these factors in early human germ cell differentiation. *GATA6* is also expressed in normal human testis, at least during fetal development. GATA6 in fetal human testis is most abundant in Sertoli and Leydig cells (92), where it may have overlapping roles with GATA4 if one transposes the comparable rodent data to humans (38).

Several mutations in either the *GATA4* or *FOG2*/*ZFPM2* genes have been identified in human cases of differences of sexual development (DSD) (96–100). While most of the reported *GATA4* and *FOG2/ZFPM2* variants appear to have a benign impact on XY DSDs (98), at least three FOG2/ZFPM2 (p.S402R; p.R260Q; p.R260Q/M544I) (97), and two *GATA4* missense variants (p.G221R; p.W228C) (96, 98), are considered to be pathogenic with reduced transcriptional activities on the human *AMH* promoter, a known GATA4 target. The fact that most of the reported variants are heterozygous in nature suggests that correct functional dosage of these transcription factors is critical for typical XY sex differentiation in humans. In support of this, haploinsufficiency of either *Gata4* or *ZFPm2*/*Fog2* is also known to induce gonadal sex-reversal on specific genetic backgrounds in mice (43).

## Conclusion

**Figure 1:**
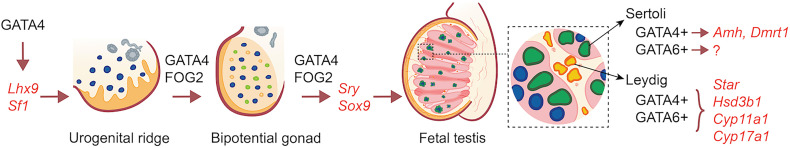
Overview of the action of GATA factors in XY gonad development and fetal testis gene expression.

GATA factors are incontrovertibly essential regulators of developmental and functional processes in multiple organs systems. The mammalian testis is no exception where at least three GATA factors (1/4/6) play essential, and sometimes overlapping roles, in controlling the initiation of male gonad development, testis differentiation at the time of sex differentiation, and gene expression and function in both the fetal and mature testis (an illustrative overview in presented in **Figure 1**). Over the years, much of our knowledge of the mechanisms of GATA action in the testis have come from genetic manipulation of the different *Gata* genes in mice and the analysis of the *cis*-regulatory regions of many of its proposed target genes in cell lines. From this point forward, the challenge will be to fine-tune this knowledge base by ascertaining not only what testis genes are regulated by GATA factors but more so, by identifying the genes that are directly targeted by these factors. The recent development and applicability of new technologies has made this a reality as we are now capable of probing the role of individual *cis*-regulatory elements in suspected genes by genome editing. Therefore, understanding GATA function in the testis is far from complete, but rather has just begun.

## Author Contributions

RV and JT drafted and edited the final version of the manuscript. KM prepared the illustration and participated in the final editing of the manuscript. All authors contributed to the article and approved the submitted version.

## Funding

Open-access fees for this publication were provided by project grant PJT-166131 from the Canadian Institutes of Health Research to RV.

## Conflict of Interest

The authors declare that the research was conducted in the absence of any commercial or financial relationships that could be construed as a potential conflict of interest.

## Publisher’s Note

All claims expressed in this article are solely those of the authors and do not necessarily represent those of their affiliated organizations, or those of the publisher, the editors and the reviewers. Any product that may be evaluated in this article, or claim that may be made by its manufacturer, is not guaranteed or endorsed by the publisher.
